# Prenatal exposure to incubation calls affects song learning in the zebra finch

**DOI:** 10.1038/s41598-018-33301-5

**Published:** 2018-10-15

**Authors:** Andrew C. Katsis, Mzuri H. Davies, Katherine L. Buchanan, Sonia Kleindorfer, Mark E. Hauber, Mylene M. Mariette

**Affiliations:** 1 Centre for Integrative Ecology, School of Life and Environmental Sciences, Deakin University, Geelong, Australia; 2 College of Science and Engineering, Flinders University, GPO Box 2100, Adelaide, 5001 Australia; 3 Department of Animal Biology, School of Integrative Biology, University of Illinois, Urbana-Champaign, IL 61801 USA

## Abstract

Songbirds are important models for understanding the mechanisms and fitness consequences of imitative vocal learning. Although the effects of early-life environmental and social conditions on song learning are well-established, the impact of early sound exposure has received surprisingly little attention. Yet recent evidence hints at auditory sensitivity in songbird embryos, including in the zebra finch (*Taeniopygia guttata*), a classic model species for song learning. Here, we tested whether prenatal exposure to incubation calls—highly rhythmic parental vocalisations produced on the nest—affected song learning in zebra finches. Embryos were exposed in the egg to either incubation (treatment) or contact (control) calls, and after hatching were reared in a large colony. The playback treatment did not affect song complexity nor the accuracy of song copying from the social father, but instead increased learning of non-paternal song syllables. This, in turn, improved males’ mounting success in mating trials. These effects may be attributable to changes in juvenile social behaviours, as playback also influenced male behaviour during mating trials. Our study provides the first experimental evidence that prenatal acoustic environment affects song learning and courtship behaviour in songbirds, thereby raising interesting questions on the role of innate versus acquired biases for vocal learning.

## Introduction

Songbirds are classic animal models for understanding the neural, social and behavioural processes of imitative vocal learning, which have many striking parallels in human speech^1^. During songbird vocal learning, individuals memorise and then reproduce tutors’ songs, and in many species this occurs during one or more ‘sensitive phases’ of development^2^. Auditory sensitivity is not considered to fully develop in altricial songbirds until the first week post-hatching^3,4^, suggesting that vocal learning could only begin after this time. However, several recent songbird studies provide evidence that embryos do respond to acoustic stimuli^5,6^, and that exposure *in ovo* to particular conspecific vocalisations, such as parental incubation calls, affects development and postnatal behaviour^7,8^, with long-lasting fitness consequences^8^. Despite this evidence, no study to date has experimentally tested if and how an embryo’s acoustic environment affects song learning.

In precocial birds and in humans, nonetheless, the effects of prenatal acoustic stimulation on early postnatal vocal discrimination and social behaviour have been recognised since the 1960s (reviewed by Bolhuis^9^). Classic and more recent imprinting studies in chickens, ducks and quails (which do not sing) show that embryos are sensitive to sound^10^, and that hatchlings are more likely to approach or respond vocally to sounds previously heard in the egg^11–13^. These findings are paralleled in a number of human studies demonstrating that newborns recognise acoustic stimuli that they experienced *in utero*^14,15^. It is, therefore, plausible that prenatal sound also alters song learning in songbirds, by predisposing individuals to respond to certain sensory stimuli over others or altering social behaviours important for song learning. Accordingly, a recent study in superb fairy-wrens (*Malurus cyaneus*) suggested that embryos responded to males’ (but not females’) songs, as indicated by changes in embryonic heart rate^6^. Likewise, in the closely-related red-backed fairy-wren (*M. melanocephalus*), a correlational study found that individuals’ adult song partly resembled their mother’s song, which in turn had similarities with her incubation call^16^. This raises the possibility that prenatal incubation call exposure might, indeed, bias individuals towards learning particular songs later in life, although this remains to be tested.

Underlying those perceptual and behavioural effects, there is evidence that prenatal acoustic stimulation also alters neural development in the auditory system and associated brain regions, with broader consequences for cognition^17,18^. Specifically, pre- and perinatal exposure to species-specific sounds, music or noise has been shown in multiple species—including chickens, rats and humans—to modify neural connectivity and plasticity in several parts of the brain^15,18,19^, including the hippocampus, and to affect performance in cognitive tasks^17,20^. Importantly, the characteristics of the acoustic stimulus are crucial for predicting its effects, with rhythmic sounds, such as music, tending to improve cognitive performance, and arrhythmic sounds, such as noise, having the opposite effect (reviewed by Chaudhury^18^).

Across taxa, rhythm seems to be an especially salient feature of vocalisations for embryos. For example, human newborns presented with speech that has been low-pass filtered (preserving its rhythm but reducing other linguistic information) prefer to hear the language that they experienced prenatally^21^, and readily distinguish between languages from different rhythmic classes but not from the same rhythmic class^22,23^. Similarly, Peking duck (*Anas platyrhynchos*) embryos and hatchlings rely heavily on calls’ temporal pattern for correct identification^24,25^. Beyond pre- and perinatal effects, rhythm may also be important for subsequent song learning. Interestingly, bobwhite quail (*Colinus virginianus*) embryos exposed to conspecific vocalisations showed cellular activation in several auditory-related brain regions, including the caudomedial nidopallium^26^—a region that, in songbirds, is activated primarily by conspecific song^27^. In the zebra finch (*Taeniopygia guttata*), an altricial songbird, ZENK expression in the caudomedial nidopallium was higher in response to rhythmic conspecific songs than to manipulated arrhythmic songs, as early as 15 days post-hatching (i.e. prior to song learning onset)^28^. We may, therefore, hypothesise that highly rhythmic stimuli heard prenatally, such as fast parental incubation calls, could have stimulatory cognitive effects that are beneficial for vocal learning, including for song learning later in life. This effect might occur regardless of whether vocal learning is the primary target of selection underlying the evolution of such parent-embryo communication.

To investigate the effects of prenatal sound exposure on song learning, and the consequences for courtship behaviours, we studied the zebra finch, a classic avian model species for vocal learning^29^. If prenatal sound does influence song learning, then the fitness implications could be substantial, given the crucial role of male song in mate attraction and consequent reproductive success^30^. Female zebra finches generally prefer songs that are more complex^31–33^, and perhaps more accurately copied from a tutor^34^, although the various approaches used to characterise song quality make it difficult to assess the relative importance of individual song traits^30^. Males preferentially learn their song from the adult male who provides the most care^35^, which is typically the father in this monogamous biparental care species^36^. The zebra finch is a closed-ended vocal learner, with a sensitive period for song learning currently established between ages 35 and 65 days^37^ and song crystallisation at about 90 days old^37^. However, zebra finches were also recently suggested to partly learn their begging call as young nestlings, implying that vocal learning may start much earlier than currently acknowledged for this species^38^. Furthermore, Mariette and Buchanan^8^ showed that zebra finch parents produce a distinct incubation call, at high temperature and during the final third of the incubation period^8^. These calls are acoustically distinctive from other zebra finch in-nest vocalisations, being high-pitched (6–10 kHz) and very rhythmic, with fast and regularly spaced calls at a rate of 5 to 6 calls/sec^8^. Experimental prenatal exposure to this call subsequently altered nestlings’ begging call behaviour, as well as their growth rate, with consequences for later reproductive success^8^. However, the neuronal and physiological mechanisms underlying this response are currently unknown. In particular, it remains to be tested whether these effects on growth rate—and later reproductive success—occur directly, or due to changes in vocalisations, including begging calls and perhaps song later in life.

Here, we experimentally exposed captive, wild-derived zebra finches *in ovo* to either conspecific incubation calls (treatment) or conspecific contact calls (control), and tested the effects of such prenatal acoustic experience on both song learning and attractiveness. We predicted that males stimulated with incubation calls would show enhanced vocal learning, leading them to produce higher quality songs at maturity, and that males with higher quality songs would experience greater courtship success in mating trials. We assessed two aspects of song quality, by measuring song acoustic parameters as a measure of song complexity, as well as quantifying song copying accuracy by comparing father and son songs based on both sonograms and syllable types. As part of the latter analysis, we also quantified offspring syllables that were absent from the songs of their social father, since the birds were reared in a large colony (rather than in isolated family groups), and therefore had access to many potential adult and juvenile tutors, mimicking a typical situation in the wild^37^. In addition, because incubation calls are higher-pitched than other zebra finch vocalisations, we predicted that males exposed to incubation calls may sing at higher frequencies, thereby advertising the prenatal environment that they encountered.

## Results

### Effects of prenatal call exposure on song parameters and complexity

For each male’s song, we measured eight acoustic parameters: song duration, number of syllables per song, number of syllable types, syllable duration, average entropy, peak frequency, and first and third quartile frequency. Treatment and control males did not differ in any acoustic parameters, when considering each parameter singly (Supplementary Table S1) or when parameters were combined into 3 principal components (PCs). Contrary to our predictions, PC1 (where frequency parameters loaded positively) was not higher for treatment males (t = 1.57, p = 0.126), and PC2 (where song duration and number of syllables, proxies for song complexity, loaded negatively) was not lower (t = −1.01, p = 0.319). Males that were heavier as nestlings produced songs with longer syllables (t = 2.05, p = 0.048), but nestling mass had no significant effect on any other acoustic parameters or PCs.

### Effects of prenatal call exposure on song learning

**Figure 1:**
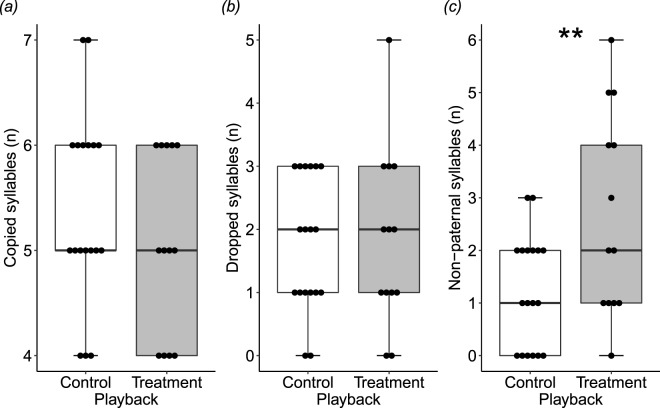
The effects of prenatal acoustic playback on song syllable learning in male zebra finches. Boxplots (with individual data overlaid) showing the effects of treatment (incubation calls, n = 13) and control (contact calls, n = 18) playback on the number of song syllables that males **(a)** copied from their father, **(b)** failed to copy from their father, and **(c)** copied from non-father males or improvised. Double asterisks (**) indicate significant difference between treatment and control males (GLMM: z = 2.78, p = 0.006).

We used two independent approaches to measure song similarity between males and their social fathers: (1) matching syllable types between fathers and sons via sonogram visual dissection, and (2) calculating father-son song similarity via automated sonogram comparison (i.e. ‘Similarity’ function in Sound Analysis Pro^39^). With the syllable matching approach, there was no effect of treatment on how many syllables sons learnt from their father (copied syllables; t = −0.82, p = 0.418) nor failed to learn from their father (dropped syllables; z = 0.27, p = 0.787) (Table 1). Interestingly, however, treatment males sang more syllables that were not present in their father’s song (non-paternal syllables; z = 2.78, p = 0.006; Table 1, Fig. 1).

**Table 1:** Output from LMMs or GLMMs testing the effects of prenatal playback of incubation calls (treatment; n = 13) or contact calls (control; n = 18) on the number of song syllables that males (a) copied from their father, (b) failed to copy from their father, and (c) copied from non-father males or improvised. Models include playback treatment, father song complexity (number of different syllable types), and son nestling mass as fixed effects; and father identity as a random effect. For the variable ‘playback treatment’, estimates and SEs are for treatment males, with control as a reference.

	Estimate	SE	T-value	P-value
**(a) Copied syllables**				
playback treatment	−0.26	0.32	−0.82	0.418
father syllable types (n)	0.38	0.16	2.41	**0.023**
nestling mass	0.04	0.16	0.26	0.796
	**Estimate**	**SE**	**Z-value**	**P-value**
**(b) Dropped syllables**				
playback treatment	0.08	0.28	0.27	0.787
father syllable types (n)	0.41	0.12	3.42	**0.001**
nestling mass	−0.01	0.14	−0.07	0.941
**(c) Non-paternal syllables**				
playback treatment	0.84	0.30	2.78	**0.006**
father syllable types (n)	−0.11	0.15	−0.76	0.447
nestling mass	−0.14	0.16	−0.88	0.380

Using Sound Analysis Pro, treatment and control offspring did not differ in their similarity to their father’s song, using any of the three similarity measures (broad-scale similarity, fine-scale similarity and sequential similarity; Table 2). If fathers sang a greater number of syllable types (i.e. had more complex songs), their sons copied their songs with lower broad-scale similarity (t = −2.53, p = 0.021), but fine-scale similarity and sequential similarity were unaffected. Males with lower nestling mass had reduced fine-scale song similarity with their fathers (t = 3.51, p = 0.002; Table 2).

**Table 2:** Output from LMMs testing the effects of prenatal playback of incubation calls (treatment; n = 13) or contact calls (control; n = 18) on three measures of father-son song similarity: (a) broad-scale (arcsine-transformed), (b) fine-scale, and (c) sequential similarity. Models include playback treatment, father song complexity (number of different syllable types), and son nestling mass as fixed effects; and father identity as a random effect. For the variable ‘playback treatment’, estimates and SEs are for treatment males, with control as a reference.

	Estimate	SE	T-value	P-value
**(a) Broad-scale similarity**				
playback treatment	−0.04	0.08	−0.46	0.650
father syllable types (n)	−0.10	0.04	−2.53	**0.021**
nestling mass	0.06	0.04	1.59	0.127
**(b) Fine-scale similarity**				
playback treatment	−1.30	0.72	−1.81	0.082
father syllable types (n)	−0.22	0.37	−0.59	0.564
nestling mass	1.24	0.35	3.51	**0.002**
**(c) Sequential similarity**				
playback treatment	−2.19	6.35	−0.35	0.733
father syllable types (n)	2.13	3.11	0.69	0.499
nestling mass	1.01	3.31	0.31	0.763

### Effects of prenatal call exposure and song learning on courtship behaviour

**Figure 2:**
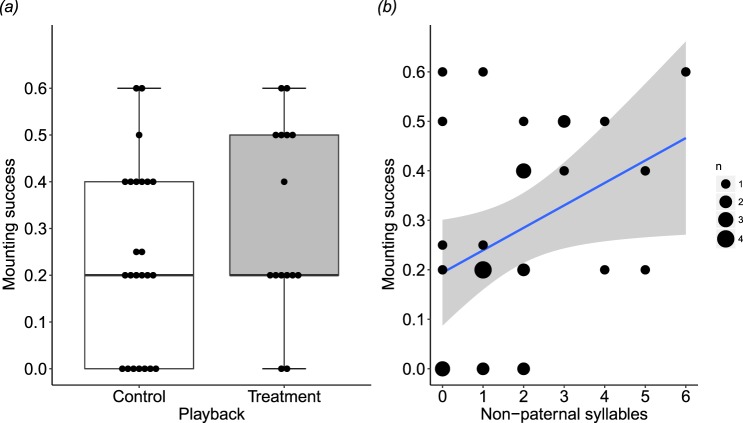
The effects of prenatal acoustic playback and song syllable learning on males’ mounting success during no-choice mating trials. (**a**) Boxplot (with individual data overlaid) showing no effect of prenatal acoustic playback on mounting success during mating trials (treatment: incubation calls, n = 15; control: contact calls, n = 24). Mounting success was calculated as the probability of mounting at least once per trial, across 4 or 5 trials per individual. (**b**) Males whose songs contained more non-paternal syllables were more likely to mount a female during mating trials (n = 31 males; GLMM: z = 1.99, p = 0.047). Larger dots represent multiple males in the same coordinates.

During mating trials, treatment males approached females significantly more than controls (z = 2.03, p = 0.042), but were no more likely to sing (z = 1.17, p = 0.244) or raise their head feathers (z = 0.20, p = 0.846) (Supplementary Table S2). There was no difference between treatment and control groups in the likelihood of mounting (z = 1.20, p = 0.230; Fig. 2) or copulation (z = 1.30, p = 0.193) (Supplementary Table S2), and nestling mass had no effect on any measure of courtship behaviour. Irrespective of playback treatment, a male’s mounting success was best predicted by the number of non-paternal syllables he learnt, compared to the other five measures of song learning (AIC = 163.9, compared to 165.0–168.0 for the other measures). This was also the only song learning measure that predicted mounting success, being just below the significance threshold (z = 1.99, p = 0.047; Table 3, Fig. 2). No such effect was detected for copulation success (z = 0.31, p = 0.757), possibly because successful copulations were rare, occurring in fewer than 10% of mating trials (18 out of 188 trials).

**Table 3:** Output from GLMMs testing the effects of song learning on the likelihood of male mounting during a mating trial, independent of prenatal playback treatment. Each model used mounting (yes/no, per trial) as the dependent variable, and one measure of song copying accuracy (n = 148 trials from 31 males) or song complexity (n = 188 trials from 39 males) as the fixed effect. Female movement (normal/abnormal) was also included as a fixed effect, with male identity and female identity included as random effects unless their parameter estimate was nil.

	Estimate	SE	Z-value	P-value
**Measure of song copying accuracy**				
copied syllables (n)	−0.36	0.27	−1.37	0.172
dropped syllables (n)	−0.12	0.22	−0.57	0.566
non-paternal syllables (n)	0.50	0.25	1.99	**0.047**
broad-scale similarity	0.22	0.21	1.07	0.286
fine-scale similarity	0.08	0.20	0.41	0.684
sequential similarity	−0.36	0.21	−1.72	0.086
**Measure of song complexity**				
total syllable types (n)	0.30	0.18	1.69	0.091

## Discussion

In this study, we provide the first experimental evidence that the prenatal acoustic environment affects song learning and courtship behaviour in a model songbird species. Rather than affecting song complexity or copying accuracy from the father as we predicted, exposing embryos to rhythmic incubation calls increased their learning of non-paternal syllables and their tendency to approach females at adulthood. Together, both of these effects suggest possible changes in males’ social behaviour. In turn, the number of non-paternal syllables was also the only song learning parameter to predict mating success (albeit weakly) in our individuals reared in complex social groups. To date, prenatal acoustic communication in birds has been shown to affect hatching time^40^, hatching success^41^, nestling begging behaviour^7,8^ and long-term microhabitat choice and fitness^8^. Our findings now extend the importance of the prenatal acoustic environment to song learning in an oscine bird.

Contrary to our expectations, enriching the prenatal acoustic environment with highly rhythmic incubation calls, in addition to other zebra finch nest vocalisations, did not improve song complexity or copy accuracy. This lack of effect appeared robust, since it was evident across both established methods we used to measure song copy accuracy (i.e. syllable matching and sonogram comparisons). These results on paternal copying accuracy are not necessarily incongruous with treatment males learning more non-paternal syllables: The Sound Analysis Pro software used to quantify song learning calculates the similarity of sounds present in both the tutor and tutee songs, with the similarity score unaffected by the addition of non-paternal syllables to the son’s song^42^. Together, our results suggest that exposure to incubation calls does not affect how well juveniles learn *per se*, but, rather, from whom they learn.

Laboratory studies of song learning often place tutees with a single tutor, or a small number of potential tutors, which may not accurately replicate the social experience of wild birds^35^. As in other studies that reared offspring in aviaries with many potential tutors^35,43^, males in our experiment (n = 24; or 77% of all sons) commonly sang syllables that were not present in their father’s song. These non-paternal syllables were either copied from other males in the colony or were novel and improvised^35^. We consider the former explanation to be likelier in most cases, as 15 out of 16 non-paternal syllable types sung by offspring were also sung by at least one other focal father in the experiment. Furthermore, in a previous song learning study with zebra finches raised in an aviary setting, syllables learnt from non-father tutors were three times more common among offspring than improvised syllables^35^. The factors driving tutor choice in juvenile birds warrant further study, given that this decision determines the song that males will then use to attract females. Our data also show that prenatal exposure to incubation calls enhances learning of non-paternal syllables, providing the first experimental evidence that very early acoustic experience can, if indirectly, affect the later refinement of the crude song template, and thus affect song learning. Our results do not provide evidence for a direct effect of early acoustic experience either on the developing neural song system nor on any innate song learning predispositions. While previous studies indicate that individuals’ preference for conspecific tutors appears to rely on innate mechanisms^44^, our results suggest that early acoustic experience may refine tutor selection within species. Whether the rhythmic nature of the incubation calls was the acoustic feature contributing to this outcome could be explicitly tested in the future, by artificially manipulating the rhythmicity of the stimuli to which embryos are exposed.

The specific mechanisms leading to treatment males learning more non-paternal syllables remain to be investigated, but we suspect changes in social behaviours may have played a role. Tutor choice is affected by social interactions, with juvenile zebra finches tending to learn from males that provide the most care^35,45^ or show them more aggression^46,47^, as well as from other juveniles (at least when held together without adults)^48^. Given the number of potential tutors in our colony, we could not identify the source of these non-paternal syllables. However, zebra finch fledglings living in both wild and captive colonies tend to form temporary crèches^37^, in which they interact with other juveniles and with unrelated parents, who have been noted to occasionally feed juveniles other than their own^35^. It is possible that exposure to incubation calls facilitated learning of non-paternal syllables by increasing juveniles’ social interactions with peers and unrelated adults during song learning. Specifically, since offspring exposed to incubation calls and high post-hatching temperatures were lighter at fledging^8^, they may have associated more strongly with other fledglings and fathers to solicit food, and thereby allow for compensatory growth. Consistent with this hypothesis, zebra finch offspring dosed with the avian stress hormone corticosterone, which also leads to lower body mass, associate more with other individuals than with their parents, and thus occupy more central positions in social networks^49^. Social experience as a juvenile can, in turn, influence adult courtship behaviour and pairing success in males zebra finches^50^. Accordingly, in our study we found a behavioural difference between treatment and control subjects, with treatment males approaching females more frequently during mating trials. This suggests that future work testing the effects of prenatal acoustic stimulation on juvenile social interactions, and their consequences for song learning, may yield insightful results.

The number of non-paternal syllables was the only of our song copying measures to be affected by the playback treatment, and also the only measure to predict mounting success (which only occurs in zebra finches with female consent)^51^. This latter effect was close to the significance threshold of α = 0.05 and, therefore, warrants further investigation through an increase in sample size, for instance. It is presently unclear how females might distinguish from which tutor a potential mate has learnt his syllables, but this may be recognised indirectly through vocal or behavioural cues^52^. For instance, offspring could preferentially acquire non-paternal syllables from high-quality males, which may then make them more attractive to females. In contrast, we found no evidence that males that sang more syllable types achieved higher mounting or copulation success, which departs from previous work showing female preference for more complex songs^31–33^. Yet, in our study, males that were heavier as nestlings learnt the fine-scale details of their father’s song more accurately, consistent with the predictions of the developmental stress hypothesis^53,54^. It might be that rearing males in complex social groups, and so permitting learning of non-paternal syllables, masks inter-male differences in song complexity. Therefore, future vocal learning experiments may benefit from raising offspring in colonies rather than artificially segregated family groups, to better understand the effect of song quality on female preferences in this model species.

Our study demonstrates that an embryo’s acoustic environment alters the outcome of song learning, which in turn may influence courtship success, in a classic songbird model species. Males exposed *in ovo* to parental incubation calls produced songs with more non-paternal syllables, and approached females more often during mating trials. We also found that males varied in how many non-paternal syllables they incorporated into their songs when reared in complex social groups, and that this affected mounting success during mating trials. Our findings, therefore, open interesting research avenues into the importance of prenatal sound for vocal learning and social behaviour in songbirds, with implications in sexual selection.

## Methods

Subjects in our study were offspring from an acoustic playback experiment previously published by Mariette and Buchanan^8^, where our breeding and playback procedures (including spectrograms) were fully described in the supplementary material. Briefly here, from December 2013 to March 2014, 61 male and 61 female zebra finches of mixed age were allowed to pair and breed freely in outdoor aviaries at Deakin University, Australia^55^. These birds were 7–9 generations derived from wild (northern Victoria, Australia) origin. Eggs were collected on the day of laying, and incubated in an artificial incubator at 37.5 °C and 60% humidity. After 9 days of incubation in a single main incubator, whole clutches were randomly allocated to one of two groups (treatment or control), each exposed to a different acoustic playback in separate experimental incubators. Treatment eggs were exposed to incubation calls, a rhythmic parental in-nest vocalisation produced in sequences at a characteristic rate of 5–6 calls/s in the final third of the incubation period^8^. Control eggs were exposed to contact (tet) calls, which are typically produced by nesting parents during incubation turnover and are uttered one or two at a time, sporadically in response to the partner’s whine and contact calls (rather than with an underlying rhythm)^56^. In addition, to ensure normal stimulation of the auditory system across all embryos, both groups were exposed to a nesting “whine” call, produced by parents in response to each other^56^.

Playback occurred via two Sennheiser HD 439 headphone speakers inside the incubators, which were connected through an amplifier (Digitech 18W) to a Zoom H4nSP audio player. All eggs sat within 5 cm of a speaker and received a playback stimulus of 63–67 dB. To ensure similar temperature and acoustic conditions between groups, eggs and sound cards were swapped daily between the two experimental incubators. Eggs were exposed to 75-minute playback sequences, looped continuously between 9:30 am and 6:30 pm, from day 10 of incubation until hatching, broadly replicating the acoustic experience during natural incubation^8^. Each playback sequence comprised randomly alternating sequences of whine calls (2 minutes each, 6 minutes total) and incubation or contact calls (5 minutes each, 20 minutes total), separated by periods of silence. The sequences used incubation and contact calls from four breeding pairs (i.e. 8 individuals), and whine calls from a different set of 6 pairs. All playback sequences conserved the natural rhythm of these vocalisations, at a regular 5–6 calls/s for incubation calls and at variable intervals ranging from 1 ms to 9 s (mean ± s.d = 1.3 ± 1.4 s) for contact calls. All playback vocalisations were previously recorded in our aviaries.

Hatchlings were returned to either their biological parents (n = 106) or foster parents (n = 63), and housed together in one of two visually but not acoustically isolated aviaries. Broods contained either treatment or control nestlings only (n = 40 treatment and 47 control chicks in non-mixed broods) or a mixture of nestlings from both groups (n = 41 treatment and 41 control chicks in mixed broods). Henceforth, regardless of biological relatedness, we will use the term ‘father’ to refer to a male’s social father, whom we expected to be the primary song tutor^43^. Whether offspring were returned to genetic or foster parents did not significantly predict any measure of father-son song similarity, with one near-significant exception: Males returned to their biological parents tended to have higher sequential song similarity than those returned to foster parents (LMM: t = −2.05, p = 0.051).

Because early nutritional stress is known to impact nestling mass and later song learning^53,54^, the nestlings’ mass was recorded on day 13 post-hatching (i.e. shortly before fledging).

### Song recording and courtship behaviour

Male offspring (n = 39 birds; 15 treatment and 24 control) were tested as adults for vocal learning and attractiveness. To record songs and observe courtship behaviour, we used a no-choice chamber, in which a male and female are placed together and allowed to physically interact^50,51,57^. The fathers’ songs were recorded from December 2014 to January 2015, and the male offspring’s songs from August to October 2015 (i.e. when offspring were 16–19 months old).

For each mating trial, males were transferred in their individual home cage to a recording room and given 5 minutes to acclimatise, before the female was introduced by hand. Females were selected randomly, except to exclude their former breeding partner and prevent males from being presented twice with the same female. Females were subject to the same prenatal playback stimuli as males, but whether they were treatment or control birds did not affect any mating trial outcome, and so this was not considered further in our analyses. During each 5-minute mating trial, we recorded male song and noted the following courtship behaviours: head feather raises (in which the male alters the position of feathers at his crest, giving the appearance of a flat head^37^), approaches (male comes within a bird-length of female), mountings (male mounts female for copulation, either successfully or unsuccessfully) and copulations^50^. We selected these five behaviours because each was significantly affected by male developmental conditions in a previous zebra finch study^50^, and trials lasted for 5 minutes because zebra finches are unlikely to copulate if they have not already done so within this period^51^. Because mounting and copulation only occur with female consent^51^, these behaviours indicate both male motivation and female acceptance. Abnormal behaviours are likely to disrupt normal courtship interactions and female responsiveness; hence, we quantified female movements and classified them as moving abnormally if they were either ‘extremely agitated’ (constantly moving in the cage, without stopping for longer than 3 seconds, and flying to the cage wall) or ‘frozen’ (sitting with body low and in a frozen position, without head movements) during the whole trial. Each male was tested on 5 consecutive days. When a male did not sing, or if his songs were obstructed by female calls, we recorded him for up to an additional 3 days the following week. All mating trial observations were performed by a single observer (M.H.D.), blind to playback treatment and to song learning performance.

### Song and statistical analysis

We obtained song recordings from all 39 male offspring. A typical zebra finch song bout comprises several repeated introductory syllables, followed by a stereotyped syllable sequence that we will refer to as a “song” (a.k.a. “song-phrase”, “verse” or “motif”) that is repeated for the duration of the bout^37^. We included only directed song^30^ in our analysis, and excluded all introductory syllables. We selected 5 songs per male, except for 3 males from which we could only record 2, 3 and 4 songs, respectively.

### Effects of prenatal call exposure on song parameters and complexity

Using the software Raven Pro: Interactive Sound Analysis 1.4^58^, we measured the following acoustic parameters: song duration (s), number of syllables per song, number of syllable types, syllable duration (s), average entropy, peak frequency (Hz), and first and third quartile frequency (Hz) (the frequencies below which 25% and 75%, respectively, of the energy in the song is contained). These parameters are defined in Supplementary Table S3. Syllable types were identified by a single observer (M.H.D.), who was blind to both treatment and father-son relationships, and were distinguished based on their frequency, duration and shape on a sonogram. Because we wished to compare each male’s full repertoire of song syllables, we counted the total number of syllable types across the male’s 5 songs to obtain one value per male. For consistency, for all other acoustic parameters we took the mean value across the male’s 5 songs^54^. In addition, in all analyses, continuous predictors were scaled to improve model fit.

To test whether exposure to incubation calls affected song parameters, we used separate linear mixed models (LMM) employing the lmer function in *lme4* package v. 1.1–13 in R v. 3.3.2. Each model included one song parameter as the response variable, and acoustic playback (treatment or control) and nestling mass (a proxy for early nutritional state)^53,54^ as fixed effects, with father identity as a random effect. We also used principal components analysis (PCA function from *ade4* package in R)^59^ to reduce these eight acoustic parameters to a smaller set of uncorrelated summary variables (PCs). We retained the first 3 PCs with eigenvalues greater than 1, which together explained 77.1% of variance (eigenvalue for PC1 = 3.07, PC2 = 1.82, PC3 = 1.27; Supplementary Table S4). These PCs were then included separately as the dependent variable in three LMMs, using the same random and fixed effects as in the single parameter models described above.

### Effects of prenatal call exposure on song learning

We used two independent approaches to measure song similarity between males and their social fathers: (1) matching syllable types between fathers and sons via sonogram visual dissection, and (2) calculating father-son song similarity via automated sonogram comparison in Sound Analysis Pro^39^. Males whose father’s song could not be recorded (n = 7) were excluded from these analyses. One additional male was excluded from father-son comparisons, as our records suggested that he was returned to the wrong nest after weighing at day 13, and hence that his social parents were incorrectly assigned. However, the outcome of the analyses did not change when this male was included, with either father as his tutor.

For the syllable matching approach, we established a list of all possible syllable types (19 in total) encountered across sons and fathers. Each son was then scored based on the number of syllables types (a) sung by both father and son (“copied syllables”); (b) sung by the father, but not the son (“dropped syllables”); and (c) sung by the son, but not the father (“non-paternal syllables”). We used three separate mixed models to test whether playback treatment affected each of these variables. “Copied syllables” was tested with an LMM. “Dropped syllables” and “non-paternal syllables” were instead more characteristic of a Poisson distribution, and so were tested with a generalised linear mixed model (GLMM; glmer function in *lme4*) with a log link function and Poisson error distribution. Each model included playback treatment, nestling mass, and number of syllable types in the father’s song (a measure of song complexity)^60^ as fixed effects, and father identity as a random effect.

To quantify father-son song similarity, we used the ‘Similarity’ function in Sound Analysis Pro, which compares matching sounds in tutor and tutee songs, and quantifies their similarity based on pitch, amplitude modulation, frequency modulation, Weiner entropy and goodness of pitch^39^. We manually adjusted the amplitude threshold for each song recording to ensure syllables were correctly defined by the software. Using default settings, we compared every son’s song with every song from its father (i.e. a total of 25 comparisons per son), with each comparison producing three estimates of song similarity: % Similarity (percentage of tutors’ sounds included in the final tutee song: “broad-scale similarity”), Accuracy (similarity of each sound shared between tutor and tutee: “fine-scale similarity”) and % Sequential (similarity in the order of sounds shared between tutor and tutee songs: “sequential similarity”). In all analyses with these three measures, to ensure similar statistical power to our syllable matching approach (estimating repertoire size; see above), for each bird we used the mean similarity across 5 songs. However, using the raw dataset gave the same results in all analyses. We tested the effects of playback treatment on broad-scale similarity, fine-scale similarity and sequential similarity using three separate LMMs, each including the same fixed and random effects as were used for the syllable matching approach. Broad-scale similarity data were arcsine-transformed prior to analysis to achieve normality.

### Effects of prenatal call exposure and song learning on courtship behaviour

To test whether playback treatment affected male courtship behaviour and mating success, we used GLMMs to analyse per-trial observational data (n = 188 trials from 39 males). If males were tested more than 5 times, only the first 5 trials were analysed. Due to logistical issues, some males (n = 7) were only observed over 4 trials. Dependent variables in these models were: the number of male approaches, and whether males raised their head feathers, sang, mounted the female, or copulated (all yes/no). Playback treatment, male nestling mass, and female movement (either “normal” or “abnormal”) were included in each model as fixed effects, and male and female identity as random effects. The number of male approaches was tested with a GLMM with a log link function and a Poisson error distribution, whereas the four binary variables were tested with a GLMM with a logit link function and a binomial error distribution.

To test which measure of song learning best predicted mating success, irrespective of playback treatment, we used GLMMs with a logit link function and binomial error distribution. Each model used the occurrence of mounting as the dependent variable, and one measure of song copying accuracy (copied syllables, dropped syllables, non-paternal syllables, broad-scale similarity, fine-scale similarity or sequential similarity; n = 148 trials from 31 males) or song complexity (total syllable types; n = 188 trials from 39 males) as a fixed effect. We used separate models for each measure of song learning, rather than a single combined model, to account for correlation between several variables. Female movement (normal/abnormal) was also included as a fixed effect. Male identity and female identity were included as random effects, unless their parameter estimate was nil.

### Ethics statement

All procedures and housing of birds for this study were approved by the Animal Ethics Committee of Deakin University (permit G29-2013), and methods were carried out in accordance with the relevant guidelines and regulations.

## Electronic supplementary material

Supplementary Tables S1-S4

Dataset 1

## Data Availability

The datasets supporting this article have been uploaded as part of the supplementary material.
