# Supplementary material for: Prenatal exposure to incubation calls affects song learning in the zebra finch
Source: Sci Rep. 2018 Oct 15;8:15232. doi: 10.1038/s41598-018-33301-5 (PMC6189107; doi:10.1038/s41598-018-33301-5)
Supplement: Supplementary file 1 — Supplementary Tables S1-S4 [file 41598_2018_33301_MOESM1_ESM.docx]

Supplementary materials for:

**Prenatal exposure to incubation calls affects song learning in the zebra finch**

Andrew C. Katsis^*^, Mzuri H. Davies, Katherine L. Buchanan, Sonia Kleindorfer, Mark E. Hauber, Mylene M. Mariette

* Corresponding author: akatsis@deakin.edu.au

**List of supplementary tables**

Supplementary Table S1. Output from LMMs testing effects of playback treatment on song parameters

Supplementary Table S2. Output from GLMMs testing effects of playback treatment on behaviour

Supplementary Table S3. Definitions for acoustic parameters used

Supplementary Table S4. Descriptive statistics for acoustic parameters and PCA output

**Supplementary Table S1.** Output from LMMs testing the effects of prenatal playback of incubation calls (treatment; n = 15) or contact calls (control; n = 24) on different song parameters. Each model also included male day 13 mass as a fixed effect (n.s. in all models, except for syllable duration: t = 2.05, p = 0.048), and father identity as a random effect. Estimates and SEs are for treatment males, with control as a reference.

| **Song Parameter** | **Estimate** | **SE** | **T-value** | **P-value** |
| --- | --- | --- | --- | --- |
| song duration (s) | 0.12 | 0.06 | 1.83 | 0.076 |
| syllables (n) per song | 0.85 | 0.60 | 1.41 | 0.169 |
| total syllable types (n) | 0.47 | 0.59 | 0.81 | 0.425 |
| syllable duration (s) | -0.003 | 0.004 | -0.61 | 0.544 |
| song peak frequency (Hz) | 113.52 | 141.12 | 0.80 | 0.427 |
| Q1 frequency (Hz) | 82.82 | 182.89 | 0.45 | 0.653 |
| Q3 frequency (Hz) | 198.23 | 128.54 | 1.54 | 0.132 |
| entropy | -0.08 | 0.12 | -0.67 | 0.507 |

**Supplementary Table S2.** Output from GLMMs testing the effects of prenatal playback of incubation calls (treatment; n = 15) or contact calls (control; n = 24) on zebra finch behaviour during a mating trial. Separate models for each behaviour included the male’s day 13 mass (n.s. in all models) and female abnormal movement as fixed effects. Male and female identity were included as random effects. ‘Approaches’ is a quantitative variable, whereas the other behaviours are qualitative (yes/no) variables. Estimates and SEs are for treatment males, with control as a reference.

| **Behaviour** | **Estimate** | **SE** | **Z-value** | **P-value** |
| --- | --- | --- | --- | --- |
| approaches | 0.39 | 0.19 | 2.03 | **0.042** |
| head feather raise | 0.13 | 0.69 | 0.20 | 0.846 |
| mount | 0.45 | 0.38 | 1.20 | 0.230 |
| copulation | 0.80 | 0.61 | 1.30 | 0.193 |
| singing | 1.27 | 1.09 | 1.17 | 0.244 |

**Supplementary Table S3.** Definitions for eight acoustic parameters used in our zebra finch song analysis.

| **Parameter** | **Definition** |
| --- | --- |
| song duration (s) | Time from the start of the first syllable to the end of the last |
| syllables (n) per song | Mean number of syllables within song |
| syllable types (n) | Total number of distinct syllable types identified across all songs |
| syllable duration (s) | Mean duration of syllables within each song |
| song peak frequency (Hz) | Frequency of highest energy for each syllable, averaged across all song syllables |
| Q1 frequency (Hz) | First quartile frequency: The frequency below which 25% of the energy in each syllable is contained, averaged across all song syllables |
| Q3 frequency (Hz) | Third quartile frequency: The frequency below which 75% of the energy in each syllable is contained, averaged across all song syllables |
| entropy | Average entropy (a measure of disorder or randomness in a sounds) for each syllable, averaged across all song syllables |

**Supplementary Table S4.** Descriptive statistics (mean ± SE) for eight acoustic parameters of zebra finch songs (n = 39 birds: 24 control, 15 treatment), and their loadings for the first three extracted principal components (PC) axes. Major loadings ( > |0.40|) shown in bold.

| **Parameter** | **Control**  **(mean ± SE)** | **Treatment (mean ± SE)** | **PC1** | **PC2** | **PC3** |
| --- | --- | --- | --- | --- | --- |
| song duration (s) | 0.66 ± 0.03 | 0.78 ± 0.06 | 0.100 | **-0.679** | -0.156 |
| syllables (n) per song | 7.11 ± 0.30 | 7.95 ± 0.55 | 0.342 | **-0.507** | 0.144 |
| total syllable types (n) | 7.00 ± 0.29 | 7.40 ± 0.55 | 0.296 | -0.283 | **0.490** |
| syllable duration (s) | 0.058 ± 0.002 | 0.058 ± 0.004 | -0.360 | -0.261 | -0.275 |
| song peak frequency (Hz) | 4561.73 ± 90.24 | 4651.86 ± 89.53 | **0.436** | 0.112 | -0.281 |
| Q1 frequency (Hz) | 3020.10 ± 109.58 | 3099.04 ± 128.43 | **0.430** | 0.231 | -0.294 |
| Q3 frequency (Hz) | 5313.40 ± 74.72 | 5503.72 ± 98.88 | **0.448** | 0.022 | **-0.413** |
| entropy | 6.35 ± 0.07 | 6.29 ± 0.10 | -0.284 | -0.259 | **-0.550** |
